# Impact of Dyslipidemia on Tear Film and Meibomian Gland Dysfunction: A Cross-Sectional Study of the Interplay between Serum Lipid Profile and Ocular Surface Health

**DOI:** 10.1155/2024/7345270

**Published:** 2024-04-30

**Authors:** José-Manuel Serrano-Morales, Noelia Álvarez-Santaliestra, María Carmen Sánchez-González, Antonio Ballesteros-Sánchez, José-María Sánchez-González

**Affiliations:** ^1^Department of Physics of Condensed Matter, Optics Area, University of Seville, Seville, Spain; ^2^Department of Ophthalmology, Clínica Novovisión, Murcia, Spain

## Abstract

**Purpose:**

To determine the relationship between dyslipidemia and dry eye disease (DED), as well as its influence on tear film and meibomian glands.

**Methods:**

This cross-sectional study included 40 patients with a mean age of 35.2 ± 13.9 years without any history of dyslipidemia. DED and serum lipid profile were evaluated after 8 hours of fasting. Patients were classified according to serum lipid levels with the following cut-off values: total cholesterol (TC) (200 mg/dl), high-density lipoprotein (HDL) (40 mg/dl), low-density lipoprotein (LDL) (130 mg/dl), and triglycerides (TG) (150 mg/dl). The relationship between serum lipid levels and DED was analyzed with the following variables: dry eye questionnaire-5 (DEQ-5), first (F-NIBUT) and average (A-NIBUT) noninvasive breakup time, tear meniscus height (TMH), lipid layer grade (LLG), conjunctival bulbar redness (CBR), and upper (U-LAMG) and lower (L-LAMG) loss area of meibomian glands.

**Results:**

Regarding tear film, patients with elevated TC and LDL levels reported significantly higher DEQ-5 scores and TMH (*P* < 0.05), while those with lower HDL levels showed significantly higher LLG (*p* < 0.05). Regarding MGD, patients with elevated TC, LDL, and TG, as well as lower HDL levels showed significantly higher L-LAMG (*p* < 0.05). HDL was correlated with LLG (*p* < 0.05), while TC was correlated with TMH (*p* < 0.05) and L-LAMG (*p* < 0.05), respectively.

**Conclusions:**

Disorders in TC, HDL, LDL, and TG levels were associated with DED, having an impact on the tear film and meibomian glands, specifically in DEQ-5 scores, LLG, and L-LAMG.

## 1. Introduction

Dyslipidemia is a metabolic disorder characterized by abnormal levels of serum lipids, such as cholesterol and triglycerides [1, 2]. This condition is a major risk factor for the development of cardiovascular disease, the leading cause of death worldwide [3]. Recent evidence suggests that dyslipidemia may also have a significant impact on the ocular surface [4–7].

The tear film is a complex and dynamic mixture of lipids, proteins, and electrolytes that covers the surface of the eye [8], playing a crucial role in maintaining the integrity of the cornea and conjunctiva [9]. The lipid layer tear film (LLTF), which is located at the outermost surface, serves as a barrier to protect the underlying layers from evaporation and environmental insults [10, 11]. The lipid layer is composed mainly of meibomian gland secretions, which contain a variety of fatty acids and wax esters [8, 12]. Dyslipidemia has been linked to alterations in the meibomian gland secretions, which may lead to a decrease in the quality and quantity of the LLTF [4, 6, 7, 13–16]. This may result in a range of ocular surface disorders, such as dry eye disease (DED), meibomian gland dysfunction (MGD), and blepharitis [9], which are characterized by symptoms, such as ocular discomfort, foreign body sensation, itching, burning, and blurred vision [17]. In addition, the altered tear film can also lead to inflammation, corneal and conjunctival damage, and even vision loss [9, 18].

The pathophysiology of the relationship between dyslipidemia and the tear film is not fully understood, but it is thought to involve a combination of factors, including changes in the activity and expression of enzymes involved in lipid metabolism, alterations in the composition of meibomian gland secretions, and inflammation [15, 19]. In addition, the systemic effects of dyslipidemia, such as oxidative stress and inflammation, may also contribute to the development of ocular surface disorders [5, 20]. MGD is a chronic pathology of the posterior eyelid characterized by terminal duct obstruction and/or qualitative/quantitative changes in glandular secretion [21]. Recently, MGD has been closely related to high cholesterol levels. [22] Kuriakose et al. [23] have reported that patients with dyslipidemia have significant differences in the components of meibomian gland secretion compared to the general population, especially in cholesterol esters. In addition, the increase in normal levels of low-density lipoprotein (LDL) seems to be the factor that favors the accumulation of cholesterol esters in the meibomian glands [24]. When LDL levels are altered, the meibum melting point changes increasing its viscosity, which leads to meibomian glands obstruction [3, 4, 13, 23, 25]. As a result, changes will be generated in the tear film and ocular surface [9, 23].

Therefore, the purpose is to investigate the potential influence of dyslipidemia on tear film and meibomian gland dysfunction. The study aims to assess the correlation between dyslipidemia, as characterized by abnormal levels of lipids in the blood, and lipid layer integrity in the tear film, which is an important factor in ocular surface health.

## 2. Materials and Methods

### 2.1. Study Design and Participants

This cross-sectional study was carried out at the Pharmacy faculty facilities of the University of Seville between October 2022 and March 2023. It fulfilled all the requirements of the Declaration of Helsinki and was approved by the Andalusia' Ethical Committee Board. Before the study, informed consent was obtained from each participant.

The inclusion criteria were as follows: (1) patients ≥18 years with 8 hours of fasting; (2) DED diagnosis according to the dry eye workshop (DEWS) II [17], meeting one of the following conditions: (2.1) dry eye questionnaire-5 (DEQ-5) score ≥6, (2.2) noninvasive tear film breakup time (NIBUT) <10 seconds, and (2.3) ocular surface staining with more than five or nine corneal or conjunctival stains, respectively; and (3) MGD diagnosis according to the international workshop on MGD [26], meeting two of the following conditions: (3.1) irregularity of the eyelid margin or mucocutaneous junction, (3.2) vascularity of the eyelid margin, (3.3) plugged or capped meibomian gland orifices, (3.4) meibomian gland atrophy, and (3.5) decreased meibum quality and quantity. The exclusion criteria included the following: (1) all systemic and ocular diseases, as well as treatments that influence DED assessment; (2) ocular surgeries; (3) contact lens use; (4) pregnant or lactating women; and (4) patients who did not understand or comprehend the informed consent.

### 2.2. Dry Eye Disease Assessment

DED symptoms and signs were evaluated with the ocular surface analyzer (OSA) (SBM Sistemi, Torino, Italy), which performs objective and noninvasive measures [27, 28]. The measurement was performed according to the OSA protocol, which was designed by Sánchez-González et al. [27] to best preserve the integrity of the tear film to avoid affecting the test results: (1) conjunctival bulbar redness (CBR), (2) lipid layer grade (LLG), (3) tear meniscus height (TMH), (4) first and average noninvasive tear film breakup time (F-NIBUT and A-NIBUT), and (5) meibomian gland analysis, which include upper and lower loss area of meibomian glands (U-LAMG and L-LAMG).

DED symptoms were assessed with the DEQ-5 online version that integrates the device. Tear film stability was automatically evaluated via the detection of F-NIBUT and A-NIBUT using a Placido disc. TMH was measured manually with an integrated caliper at the intersection of the center of the pupil with the lower eyelid. To assess LLG, the lipid layer interferometric pattern was compared to the Guillon pattern [29]. CBR was detected by the device through the blood vessel fluidity of the conjunctiva and classified according to the Efron scale [30]. Meibomian gland analysis was performed on the upper and lower eyelids using infrared light. The device automatically analyzed the meibomian glands, obtaining U-LAMG and L-LAMG with a value between 0% (no glandular dropout) and 100% (the highest level of glandular dropout). All measurements were performed by an experienced and trained optometrist (JMSG) who obtained focused pictures with minimal areas of glare, which are areas of increased brightness in the picture that may produce errors in the measurement. The average of 3 measures was obtained for F-NIBUT, A-NIBUT, and TMH, while for the rest of the variables, only 1 measure was obtained. In addition, the OSA was always located in the same examination room during the study to control fluctuations in temperature and airflow.

### 2.3. Serum Lipid Profile

The point-of-care Cobas b 101 system (Roche Diagnostics, Mannheim, Germany) was used to measure serum lipid profile after fasting for at least 8 hours. According to the manufacturer's performance evaluation report [31], this system met the National Cholesterol Education Program (NCEP) guidelines for measuring lipids [32]. The lipid panel test disc was performed to quantitatively determine serum lipid parameters, such as total cholesterol (TC), high-density lipoprotein (HDL), low-density lipoprotein (LDL), and triglycerides (TG). In addition, patients were classified into 8 groups according to serum lipid levels with the following cut-off values: TC (200 mg/dl), HDL (40 mg/dl), LDL (130 mg/dl), and TG (150 mg/dl) [33]. LDL was calculated with the Friedewald formula as “LDL = TC − HDL − TG/5” [34].

### 2.4. Statistical Analysis

Data were analyzed using the SPSS statistics software, version 25.0 (IBM Corporation, Armonk, NY). Sample size calculation was estimated using the GRANMO calculator, version 7.12 (Municipal Institute of Medical Research, Barcelona, Spain). For this study, sample size calculation was based on limited literature reporting MGD [4, 6, 35]. The sample size calculation assumed a statistically significant paired difference at a 95% confidence interval and a power of 80%. An estimated sample size of 37 participants was required, considering a dropout rate of 10%. Before the analysis, the average data from both eyes were calculated. Differences between DED parameters according to serum lipid levels were analyzed with the unpaired Student's *t*-test (parametric) or Mann–Whitney's *U* test (nonparametric). The Pearson's (parametric) or Spearman's Rho correlation coefficient (nonparametric) was used to analyze the correlations between DED and dyslipidemia. Continuous variables were displayed as the mean ± standard deviation (SD) with interquartile ranges (IQRs), while ordinal categorical variables were expressed as frequencies (*n*) and percentages (%). The level of significance was *P*  <  0.05 for all comparisons.

## 3. Results

A total of 40 patients, 19 (47.5%) men and 21 (52.5) women with a mean age of 35.2 ± 13.9, were included in the study. Lipid abnormalities levels were found in the respective numbers of patients: 15 (37.5%) patients with TC >200 mg/dl; 9 (22.5%) patients with HDL <40 mg/dl; 15 (37.5%) patients with LDL >130 mg/dl; and 18 (45%) patients with TG >150 mg/dl. More details on the characteristics of the analyzed population are outlined in Table 1. The distribution of these characteristics was found to be not normal and no significant differences between groups were found in terms of age and sex.

**Figure 1:**
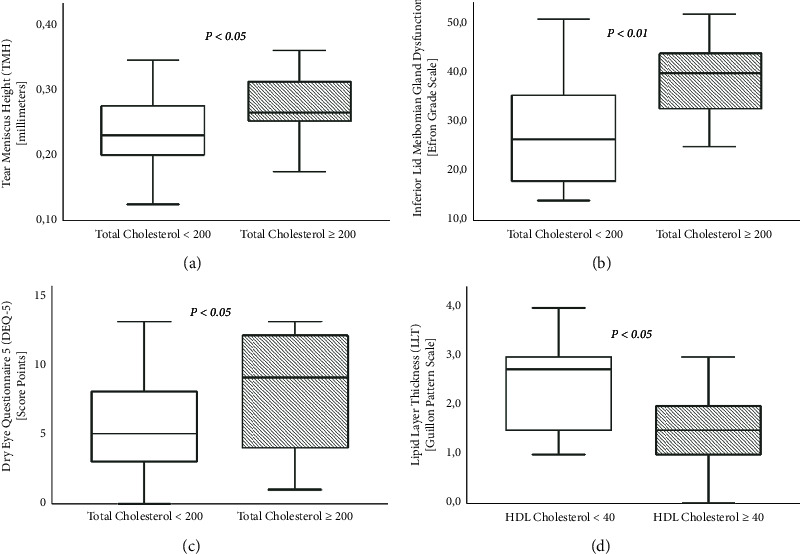
Box and whisker plots representing the association between dyslipidemia and DED symptoms and signs: (a) TMH for patients with low (<200 mg/dl) (left) and high (>200 mg/dl) (right) TC levels, (b) L-LAMG for patients with low (<200 mg/dl) (left) and high (>200 mg/dl) (right) TC levels, (c) DEQ-5 scores for patients with low (<200 mg/dl) (left) and high (>200 mg/dl) (right) TC levels, and (d) LLG for patients with low (left) (<40 mg/dl) and high (right) (>40 mg/dl) HDL levels. Each plot depicts the median, quartiles, and outliers for each group. DEQ-5: dry eye questionnaire-5; HDL: high-density lipoprotein; L-LAMG: lower loss area meibomian glands; LLG: lipid layer grade; TC: total cholesterol; and TMH: tear meniscus height.

**Figure 2:**
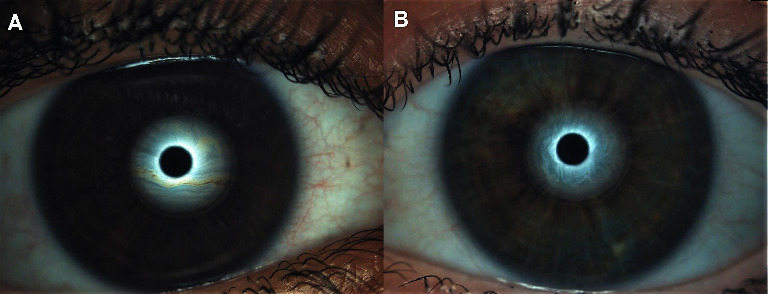
LLTF patterns in patients according to their HDL levels: (A) LLTF pattern (grade 2) for a patient with low HDL levels (<40 mg/dl) and (B) LLTF pattern (grade 1) for a patient with high HDL levels (>40 mg/dl). LLTF: lipid layer tear film and HDL: high-density lipoprotein.

**Figure 3:**
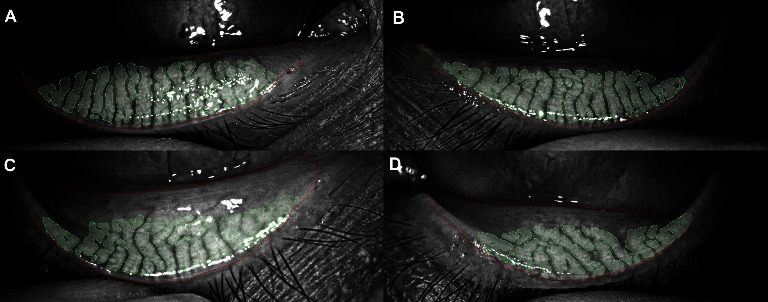
L-LAMG differences between patients according to their TC levels: (A) right (L-LAMG 12.4%) and left, (B) (L-LAMG 10.2%) lower eyelids with low TC levels (< 200 mg/dl). (C) right (L-LAMG 35.8%) and left, and (D) (L-LAMG 30.7%) lower eyelids with high TC levels (>200 mg/dl). L-LAMG: lower loss area meibomian glands and TC: total cholesterol.

The relationship between serum lipid levels and DED is outlined in Table 2. Regarding DED symptoms, a marginally significant trend in DEQ-5 scores was observed, in which patients with elevated TC (>200 mg/dl) and LDL levels (>130 mg/dl) reported more severe DED symptoms (*p* < 0.05) (Figure 1(c)). Regarding DED signs, patients with elevated TC and LDL levels also reported significantly higher TMH (*p* < 0.05) (Figure 1(a)), while lower HDL levels (<40 mg/dl) showed significantly higher LLG (*p* < 0.05) (Figures 1(d) and 2). In addition, patients with elevated TC (Figures 1(b) and 3), LDL, and TG levels (>150 mg/dl), as well as lower HDL levels reported significantly higher L-LAMG than those with lower serum lipid values (*p* < 0.05). Regarding correlations, significant positive correlations were found between TC and TMH (*r* = 0.35, *p* < 0.05) and TC and L-LAMG (*r* = 0.41, *p* < 0.05), while HDL and LLG showed significant negative correlation (*r* = −0.32, *p* < 0.05).

## 4. Discussion

Dyslipidemia is a term that represents an abnormal lipid value in one or more of the lipid profiles [2, 36]. Several studies have investigated the association between dyslipidemia and DED owing to MGD [1, 3–6, 13, 14, 16, 25, 35, 37]. However, this relationship remains unclear [7, 20]. Therefore, this study aims to analyze the relationship between dyslipidemia and DED, as well as its potential influence on tear film and meibomian gland dysfunction.

In this study, patients with elevated TC, LDL, and TG levels reported higher DED symptoms and atrophy of the lower meibomian glands than those with lower serum lipid levels. Similar results have been reported by previous case-control studies. Dao et al. [4] reported that patients with moderate to severe MGD had a higher incidence of dyslipidemia than the healthy population. Braich et al. [37] also reported that Indian patients with MGD had higher serum lipid levels than those without MGD. Furthermore, Pinna et al. [25] and Irfan et al. [38] reported similar results in young and middle-aged patients with MGD without any history of dyslipidemia. There are different theories that may explain these results. First, it is postulated that increased cholesterol may play a role in the pathogenesis of MGD [23]. Some studies have reported that the meibum of MGD patients has different components and proportions of cholesterol compared to the meibum of the healthy population [15, 19, 23]. Cholesterol is an organic substance whose melting point is around 148°C due to its larger side chains [39]. This concept may explain why the elevated cholesterol in the meibum increases its melting point compared to the normal meibum melting point, which ranges from 30°C to 34°C [22]. Theoretically, the increase in the meibum melting point makes it more viscous at physiologic temperatures leading to meibomian gland obstruction [3, 4, 13, 25, 37], which may alter tear film stability and increase its osmolarity, resulting in DED symptoms and signs [9, 37]. Second, some studies also suggest that serum lipid levels play a role in meibogenesis [7, 40]. Therefore, it is hypothesized that abnormal serum lipid levels may affect the meibogenesis in acinar cells of meibomian glands, as well as the final meibum composition leading to MGD [7]. However, some studies have also reported that elevated TC, LDL, or TG levels were not associated with DED owing to MGD [41–43].

Interestingly, this study also reported that patients with lower HDL levels showed higher atrophy of the lower meibomian glands than those with elevated HDL values. Similar results have been reported by previous case-control studies. Rathnakumar et al. [14] reported that lower HDL levels were more prevalent in patients with DED compared to those without DED. In addition, Jasmine Mary et al. [44] also reported lower levels of HDL along with the association between dyslipidemia and MGD. These results suggest that elevated HDL levels may have a positive effect on DED owing to MGD. In addition, it is well known that HDL has a preventive effect in cardiovascular disease [45, 46]. Therefore, elevated HDL levels could be beneficial for health and are not usually associated with pathologic states [20]. However, some studies have also shown that elevated HDL levels may be related to DED owing to MGD [25, 37, 42]. It is also interesting to mention why this study has obtained significant results in the lower meibomian glands, but not in the upper meibomian glands. Some studies have reported that the lower meibomian glands have a higher atrophy than the upper meibomian glands [47, 48], which may be explained by their anatomic position. Therefore, it is possible that when serum lipid levels are altered, the lower meibomian glands tend to atrophy more than the upper meibomian glands.

Regarding tear film, this study has reported opposite results. Although elevated TC, LDL, or TG levels seem to be associated with DED owing to MGD, patients with these serum lipid levels reported higher TMH values. However, patients with elevated HDL, which seems to have a protective effect against DED owing to MGD, reported lower LLG values. Therefore, the variability of these results in the associations between serum lipid levels and tear film may reflect the dyslipidemia and DED complexity [16].

### 4.1. Strengths and Limitations

To the best of our knowledge, this is the first study to investigate the potential influence of dyslipidemia on tear film. In addition, patients without comorbidities have been included to better reflect the relationship between dyslipidemia and DED owing to MGD. However, there are some limitations that may have influenced the results. First, the relatively small sample size, which was in part due to the strict enrollment criteria used to avoid possible confounders. Second, due to the cross-sectional study design, the results only suggest an association between dyslipidemia and DED. Therefore, prospective long-term controlled studies are needed to determine the potential influence of dyslipidemia on tear film and meibomian glands establishing a causal relationship, as well as the possible treatments of dyslipidemia and its effects on DED symptoms and signs. Finally, although the OSA device performs objective and noninvasive tests, observer participation is required in the measurement of TMH and LLG. Specifically, the calliper method of the OSA was used to measure TMH, which may influence the results. Therefore, further studies analyzing TMH and LLG through devices that obtain them automatically are needed [49].

## 5. Conclusions

In conclusion, this study suggests that dyslipidemia may have an impact on the tear film and meibomian glands. Elevated TC and LDL levels are associated with higher DEQ-5 scores and TMH values, whereas lower HDL values are related to higher LLG. In addition, patients with elevated TC, LDL, and TG, as well as lower HDL levels reported higher L-LAMG. However, further studies are needed in order to confirm the underlying mechanism in the association between dyslipidemia and DED symptoms and signs. Meanwhile, eye care specialists may increase their role for the early detection of dyslipidemia in patients with DED due to MGD to ensure comprehensive eye care to prevent cardiovascular disease.

## Figures and Tables

**Table 1:** Characteristics of the study population after 8 hours of fasting.

Characteristics	*n* = 40
*Demographics, mean* *±* *SD, IQR, or n (%)*	
Age (years)	35.2 ± 13.9 [18–58]
Sex, male/female	19 (47.5)/21 (52.5)
Height (cm)	170.7 ± 8.1 [156–188]
Weight (kg)	73.6 ± 14.7 [50–120]
Race (Caucasian)	40 (100)

*Serum lipids levels, mean* *±* *SD, and IQR*	
TC (mg/dl)	187.9 ± 43.2 [105–256]
HDL (mg/dl)	54.4 ± 16.2 [30–86]
LDL (mg/dl)	110.5 ± 42.1 [53–185]
TG (mg/dl)	134.7 ± 76.9 [50–400]

*Dry eye assessment, mean* *±* *SD, and IQR*	
DEQ-5, mean ± SD^a^	6.3 ± 4.1 [0–13]
F-NIBUT (s)	7.1 ± 4.6 [2.3–16]
A-NIBUT (s)	14.4 ± 9.6 [6.7–38.2]
TMH (mm)	0.2 ± 0.1 [0.1–0.6]
LLG^b^	1.8 ± 0.9 [0–4]
CBR^c^	1.8 ± 0.5 [0–2.5]
U-LAMG (%)	26.3 ± 8.1 [10.5–38]
L-LAMG (%)	32.9 ± 13 [14–73.5]

A-NIBUT, average noninvasive tear breakup time; CBR, conjunctival bulbar redness; DEQ-5, dry eye questionnaire-5; F-NIBUT, first noninvasive tear breakup time; HDL, high-density lipoprotein; IQR, interquartile range; LDL, low-density lipoprotein; LLG, lipid layer grade; SD, standard deviation; L-LAMG, lower loss area of meibomian glands; TC, total cholesterol; TG triglycerides; TMH, tear meniscus height; U-LAMG, upper loss area of meibomian glands. ^a^Values from 0 to 22. ^b^Values from 0 to 5. ^c^Values from 0 to 4.

**Table 2:** Differences between dry eye variables and serum lipid levels.

Variables^a^	TC (mg/dl)			HDL (mg/dl)			LDL (mg/dl)			TG (mg/dL)		
	Low (*n* = 25) (≤200)	High (*n* = 15) (>200)	*P value*	Low (*n* = 9) (≤40)	High (*n* = 31) (>40)	*P value*	Low (*n* = 25) (≤130)	High (*n* = 15) (>130)	*P value*	Low (*n* = 22) (≤150)	High (*n* = 18) (>150)	*P value*
DEQ-5 (0–22)	5.3 ± 3.7 [0–13]	7.9 ± 4.4 [1–13]	**<0.05** ^*∗*^	7.2 ± 4.2 [2–13]	5.9 ± 4.1 [0–13]	0.45	5 ± 3.5 [0–13]	7.9 ± 4.4 [1–13]	**<0.05** ^*∗*^	5.2 ± 3.6 [0–13]	7.5 ± 4.5 [1–13]	0.14
F-NIBUT (s)	7.1 ± 4.5 [2.2–16]	7.1 ± 5 [3.8–15.9]	0.69	4.1 ± 0.4 [3.8–4.7]	7.7 ± 4.8 [2.3–16]	0.06	6.9 ± 4.5 [2.3–16]	7.1 ± 5 [3.8–15.9]	0.76	7.1 ± 4.6 [2.3–16]	7.2 ± 4.9 [3.7–15.9]	0.56
A-NIBUT (s)	14.2 ± 9.3 [6.7–38.2]	14.7 ± 10.5 [7.1–38.1]	0.93	9.9 ± 1.9 [7.1–14.4]	15.9 ± 10.7 [6.7–38.2]	0.36	14.4 ± 9.5 [6.7–38.2]	14.7 ± 10.5 [7.1–38.1]	0.85	14.9 ± 9.7 [6.7–38.2]	13.8 ± 9.8 [6.7–38.1]	0.86
TMH (mm)	0.25 ± 0.1 [0.1–0.6]	0.27 ± 0.1 [0.2–0.4]	**<0.05** ^*∗*^	0.27 ± 0.1 [0.2–0.3]	0.25 ± 0.1 [0.1–0.6]	0.19	0.24 ± 0.1 [0.1–0.6]	0.27 ± 0.1 [0.2–0.3]	**<0.05** ^*∗*^	0.25 ± 0.09 [0.1–0.6]	0.27 ± 0.1 [0.2–0.3]	0.10
LLG (0–5)	1.7 ± 0.9 [0–4]	1.9 ± 0.8 [1–3.5]	0.58	2.4 ± 1.1 [1–4]	1.6 ± 0.8 [0–4]	**<0.05** ^*∗*^	1.7 ± 1 [0–4]	1.9 ± 0.9 [1–3.5]	0.57	1.8 ± 1.04 [0–4]	1.8 ± 0.8 [1–3.5]	0.73
CBR (0–4)	1.7 ± 0.5 [1–2.5]	1.9 ± 0.6 [0–2.5]	0.24	1.80 ± 0.67 [0.0–2.5]	1.75 ± 0.46 [1.0–2.5]	0.52	1.7 ± 0.5 [1–2.5]	1.9 ± 0.6 [0–2.5]	0.29	1.7 ± 0.5 [1–2.5]	1.8 ± 0.6 [0–2.5]	0.56
U-LAMG (%)	26.7 ± 6.9 [11–38]	25.9 ± 9.6 [10.5–38]	0.91	23.3 ± 8.9 [10.5–35]	28.1 ± 7.3 [11–38]	0.30	26.7 ± 6.9 [11–38]	25.9 ± 9.6 [10.5–38]	0.91	26.9 ± 6.9 [11–38]	25.9 ± 9.6 [10.5–38]	0.91
L-LAMG (%)	27.7 ± 9.9 [14–51]	41.5 ± 13.3 [25–73]	**<0.001** ^*∗*^	40.7 ± 15.2 [16.5–73.5]	30.3 ± 11.3 [14–64]	**<0.05** ^*∗*^	28.1 ± 9.9 [14–51]	41.5 ± 13.3 [25–73.5]	**<0.01** ^*∗*^	27.9 ± 10.23 [14–51]	38.9 ± 13.7 [18–73.5]	**<0.01** ^*∗*^

A-NIBUT, average noninvasive tear breakup time; CBR, conjunctival bulbar redness; DEQ-5, dry eye questionnaire-5; F-NIBUT, first noninvasive tear breakup time; HDL, high-density lipoprotein; LDL, low-density lipoprotein; LLG, lipid layer grade; L-LAMG, lower loss area of meibomian glands; TC, total cholesterol; TG, triglycerides; TMH, tear meniscus height; U-LAMG, upper loss area of meibomian glands. ^a^Expressed as mean ± standard deviation (SD) with interquartile ranges (IQRs). All statistically significant values are specified in bold. ^*∗*^Statistically significant level with the U Mann–Whitney test (*p* < 0.05).

## Data Availability

The datasets used and/or analyzed during the current study are available from the corresponding author on reasonable request.
